# Randomised Controlled Trials May Underestimate Drug Effects: Balanced Placebo Trial Design

**DOI:** 10.1371/journal.pone.0084104

**Published:** 2014-01-08

**Authors:** Karen Lund, Lene Vase, Gitte L. Petersen, Troels S. Jensen, Nanna B. Finnerup

**Affiliations:** 1 Danish Pain Research Center, Aarhus University Hospital, Aarhus, Denmark; 2 Department of Psychology, School of Business and Social Sciences, Aarhus University, Aarhus, Denmark; The University of Tokyo Hospital, Japan

## Abstract

**Background:**

It is an inherent assumption in randomised controlled trials that the drug effect can be estimated by subtracting the response during placebo from the response during active drug treatment.

**Objective:**

To test the assumption of additivity. The primary hypothesis was that the total treatment effect is smaller than the sum of the drug effect and the placebo effect. The secondary hypothesis was that non-additivity was most pronounced in participants with large placebo effects.

**Methods:**

We used a within-subject randomised blinded balanced placebo design and included 48 healthy volunteers (50% males), mean (SD) age 23.4 (6.2) years. Experimental pain was induced by injections of hypertonic saline into the masseter muscle. Participants received four injections with hypertonic saline along with lidocaine or matching placebo in randomised order: A: received hypertonic saline/told hypertonic saline; B: received hypertonic saline+lidocaine/told hypertonic saline; C: received hypertonic saline+placebo/told hypertonic saline+pain killer; D: received hypertonic saline+lidocaine/told hypertonic saline+pain killer. The primary outcome measure was the area under the curve (AUC, mm^2^) of pain intensity during injections.

**Results:**

There was a significant difference between the sum of the drug effect and the placebo effect (mean AUC 6279 mm^2^ (95% CI, 4936–7622)) and the total treatment effect (mean AUC 5455 mm^2^ (95% CI, 4585–6324)) (P = 0.049). This difference was larger for participants with large versus small placebo effects (P = 0.015), and the difference correlated significantly with the size of the placebo effect (r = 0.65, P = 0.006).

**Conclusion:**

Although this study examined placebo effects and not the whole placebo response as in randomised controlled trials, it does suggest that the additivity assumption may be incorrect, and that the estimated drug effects in randomised controlled trials may be underestimated, particularly in studies reporting large placebo responses. The implications for randomised controlled trials and systematic reviews need to be discussed.

## Introduction

Double-blind randomised controlled trials (RCTs), systematic reviews, and meta-analyses of RCTs are considered to be the gold standard for evidence-based treatment guidelines [1]. An inherent assumption of a meta-analysis is that the difference between the observed drug response and the observed placebo response in RCTs is the pharmacological effect of the drug. This “additive model”, although never proved, has been a general assumption of meta-analyses of drug trials since their introduction in the 1940 s [2]. The validity of this model has recently been questioned [3]–[6]. If the model is incorrect, RCTs and meta-analyses fail to give valid information about drug effect sizes and particularly about relative effect sizes across different drugs and conditions.

Several lines of evidence suggest that the additivity assumption is incorrect and that estimated effect sizes are not independent of placebo responses. First, reviews of, e.g., ulcer, depression, and pain treatment have shown that trials are more likely to be positive and have larger estimated drug effect sizes if placebo responses are smaller [7]–[10]. Second, in studies with very large placebo responses, a ceiling effect is likely to occur. This makes it impossible to show the superiority of a drug and to estimate the drug effect [11]. Third, factors contributing to the response following placebo treatment may be different from those contributing to the response following drug treatment as seen in trials of depression and menopausal symptoms [12], [13]. Along these lines, placebos, antidepressants, and analgesics have been shown to exert different effects on the brain [14], [15], so indirect evidence suggests that drug and placebo responses are only partly additive. This issue has, however, never been thoroughly investigated, although it may have serious implications for clinical trial methodology and evidence-based medicine [5], [16]–[18].

The changes encountered during placebo treatment in a standard RCT, here termed the placebo response [19], are the result of the placebo effect itself, but they may also be attributed to spontaneous remission of symptoms (the natural history), regression towards the mean, co-intervention, and patient and doctor reporting biases [20]. Thus, the placebo effect, which is defined as the difference between the changes that occur with and without the administration of a placebo, is only a part of the placebo response seen in standard RCTs [19]. In this study, the drug effect is defined as the “true pharmacological effect” of a drug (i.e. the effect of a drug when given without knowledge and thus without placebo effect), and the total treatment effect is defined as the effect of a drug given in full view of the participant (i.e. including the drug and the placebo effect).

The overall aim of the present study was to test the possible additivity of drug and placebo analgesic effects. Although the placebo effect is only part of the placebo response, it may give some suggestion of the additivity assumption of drug effects and placebo responses in RCTs. For ethical reasons, the population was healthy volunteers of at least 18 years of age. The intervention and the comparator were lidocaine and placebo given open and hidden, and the outcome was pain intensity. The study design was a balanced placebo trial design (BPTD) [21]. The BPTD is well recognised for its use in alcohol and nicotine research for studying the expectancy effect. In the within-subject BPTD, the participants receive a placebo twice and a drug twice, and the information given (correct or false) is balanced with the administration of treatments (drug or placebo). The BPTD allows estimation of the drug effect (when participants are given a drug without knowledge), the placebo effect (when given a placebo and told drug), and the total treatment effect (when given a drug and told drug), and thus allows assessment of additivity. We chose a pain model with intramuscular injection of hypertonic saline because it produces a consistent moderate pain that can be reliably reduced by co-administration of lidocaine and because it provides a method for giving the drug (lidocaine) in full view of the participant (open) and without the participant’s knowledge (hidden) [22], [23]. The primary hypothesis was that the total treatment effect is smaller than the sum of the drug effect and the placebo effect. We also hypothesised that the difference between the total treatment effect and the sum of the drug effect and the placebo effect is larger in participants with large placebo effects than in those with small or no placebo effects, and that this difference is positively correlated with the magnitude of the placebo effect. We found a significant placebo effect, and the design was therefore suitable for testing our hypotheses.

## Methods

### Study Design

The study applied a within-subject balanced placebo trial design [24] in an experimental jaw muscle pain model in healthy individuals.

### Participants

Healthy volunteers who were at least 18 years old were recruited through advertisement at educational institutions. Exclusion criteria were chronic pain, inability to cooperate, psychiatric or neurological disorders, previous significant problems in teeth or jaw, diabetes, significant medical conditions, allergy to lidocaine, pain on examination days, intake of pain medication during the past week, alcohol and drug abuse, and previous participation in trials using the same method (injection of hypertonic saline into the masseter muscle). All participants received financial compensation for their participation in the study.

The experiment was carried out at the Danish Pain Research Center, Aarhus University Hospital, Denmark. Inclusion of the participants took place from 7 March to 12 October 2012. The last follow up took place on 21 November 2012. Approval was given by the Ethical Committee of the Central Denmark Region (no. 1-10-72-114-12) and the Danish Data Protection Agency (no. 1-16-02-19-12), and all participants signed an informed consent document at inclusion. As the purpose of the study was not to evaluate an effect of an intervention, it is not a clinical trial and has only been registered with the ethical committee. Participants were informed that the aim of the study was to investigate the variability of pain intensity by means of an experimental pain model with and without concomitant analgesic treatment.

### Pain Model

Pain was induced by injection of hypertonic saline (HS, 5% in 0.2 ml) into the deep central part of the masseter muscle using a 1 ml syringe with a disposable 27G stainless needle [22], [23]. Pain intensity was continuously rated on an 100-mm electronic visual analogue scale (eVAS), where 0 mm indicated “no pain” and 100 mm “worst pain imaginable”, until pain had subsided completely [22]. Pain intensities were sampled every second by the computer.

### Intervention

We used lidocaine (1%) as analgesic treatment. Lidocaine 2% administered concomitantly with HS provides almost complete pain relief [22], and in pilot studies we determined the dose necessary to provide approximately 50% pain relief for the purpose of the present study. The addition of lidocaine does not give rise to side effects, numbness, or other sensations that may unblind the participants.

### Conditions and Manipulations

The study consisted of two separate sessions: 1) A pre-experimental session where all participants received one injection with HS. In order to estimate the placebo effect, it is necessary to have a relatively large inter-individual variability in placebo effect size. Half of the participants were therefore conditioned with lidocaine because this has been shown to increase the magnitude of the placebo effect [25], [26], while the other half were not conditioned. 2) An experimental session where the drug effect, the placebo effect, and the total treatment effect were tested. The same investigator (KL) performed all the examinations, and the participants were placed in a hospital bed in a supine position in all sessions.

In the pre-experimental session, participants were randomised to either no conditioning or conditioning using a simple computer-generated randomisation list (http://www.randomization.com/). Participants in the no conditioning group received one injection with HS (0.1 ml HS 10% mixed with 0.1 ml sterile water, which yielded a concentration of HS 5% in 0.2 ml). Participants in the conditioning group received one injection with HS (HS 5% in 0.2 ml) and one injection with HS and lidocaine (0.08 ml HS 10% mixed with 0.12 ml lidocaine 1%, which yielded a total volume of 0.2 ml) in order to give them an experience of pain relief when they were given the same drug in the experimental session.

**Figure 1:**
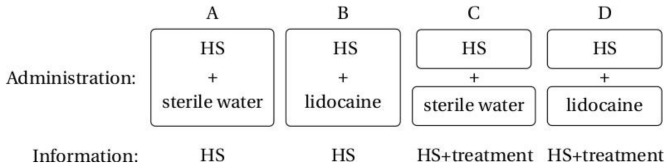
Experimental setup.

In the experimental session, participants received four injections in randomised order using a simple computer-generated randomisation list. The randomisation list was generated by a person who was not involved in the study. When a new sequence was assigned, it was revealed to the study nurse who prepared the vials, but was otherwise not involved in the study. The allocation was blinded to the investigator (KL), who enrolled participants and assigned them to interventions, but was blinded to the type of intervention. The allocation sequence was concealed until all participants had completed the study and the database was closed. Two of the injections included lidocaine (0.1 ml HS 10% mixed with 0.1 ml lidocaine 1%): one was open (D) and one was hidden (B) (Table 1, Figure 1). The two remaining injections (A,C) were with HS and matching placebo (0.1 ml HS 10% mixed with 0.1 ml sterile water): one of which was mixed in full view of the participants (C) (Table 1, Figure 1).

**Table 1:** Study design.

		Information	
		No drug	Drug
**Administration**	**No drug**	A - Control	C - Placebo
	**Drug**	B - Drug	D - Total

The order of injections was randomised.

The following injections and verbal suggestions were given to each participant in randomised order: Injection A: HS was given and the participant was told: ”You will now receive an injection with saline that produces experimental muscle pain” (control condition). Injection B: HS with lidocaine was given and the participant was told: ”You will now receive an injection with saline that produces experimental muscle pain” (drug condition). Injection C: HS was mixed with sterile water in full view of the participant who was told: “Now I will mix the saline with a potent pain killer” (placebo condition). Injection D: HS was mixed with lidocaine in full view of the participant who was told: “Now I will mix the saline with a potent pain killer” (total treatment condition). It was emphasised to the participants that injections with the same concentration of saline are known to produce pain of various intensity depending on the precise site of injection.

The investigator was blinded to whether the injection (A/B) or the vial added to the HS (C/D) was with or without lidocaine. To ensure blinding, the two vials (C/D) and the injections A and B were prepared beforehand by a study nurse who was not involved in the study using the randomisation list, and they were identical in appearance. All participants received the first and the second injection in the left and right masseter muscle, respectively. The third and the fourth injection were administered in the right and left masseter muscle, respectively. There was a short break between the first and the second injection and the third and the fourth injection, and no injections were given before the pain from the previous injection had completely disappeared. A 2-hour break was interposed between the second and the third injection. During the break, the participants rested in a waiting room. Pilot studies showed that participants reported normal sensation and had normal sensation to pinprick stimuli 1–1.5 hour after an injection with HS and lidocaine, and we thus expected a minimum carry-over effect from lidocaine.

Data is available upon request to the corresponding author.

### Outcomes and Statistical Analyses

The effects were defined as follows: The drug effect (δ) was the difference in pain between the control (A) and the drug (B) condition. The placebo effect (μ) was the difference in pain between the control (A) and the placebo (C) condition. The total treatment effect (γ) was the difference in pain between the control (A) and the total treatment (D) condition.

The predefined primary purpose was to examine whether there was a difference between the total treatment effect (γ) and the sum of the drug effect and the placebo effect (δ+μ). The predefined secondary purpose was to examine whether this difference (γ-(δ+ μ)) depended on the placebo effect and thus a) to compare this difference between those with large and small placebo effects and b) to test if there was a correlation between this difference and the placebo effect. The predefined primary outcome measure was the area under the curve (AUC, mm^2^) of the pain intensity during the injections. The secondary outcome measure was peak pain (PP, mm). Statistical analyses were performed using the statistical software STATA, version 12, and SPSS, version 20. Considering a difference in the AUC of at least 800 mm^2^ and a standard deviation (SD) of 1800 of the difference for each subject, 42 participants were expected to be sufficient to obtain a statistical power exceeding 80% (α = 0.05). Data were checked for normality using histograms and Q-Q plots. Results are described with mean, SD, or 95% confidence intervals (CI). Continuous data with a normal distribution were analysed with paired and unpaired t-tests. Correlation was assessed using Pearson’s correlation. P<0.05 was considered statistically significant. Multiple comparison analysis of the four treatments was included as a post-hoc analysis performed with three-way analysis of variance (ANOVA) with AUC and PP as the dependent variable and the treatments (A, B, C, or D), subject, sequence, and first-order carry-over effect as factors.

## Results

**Figure 2:**
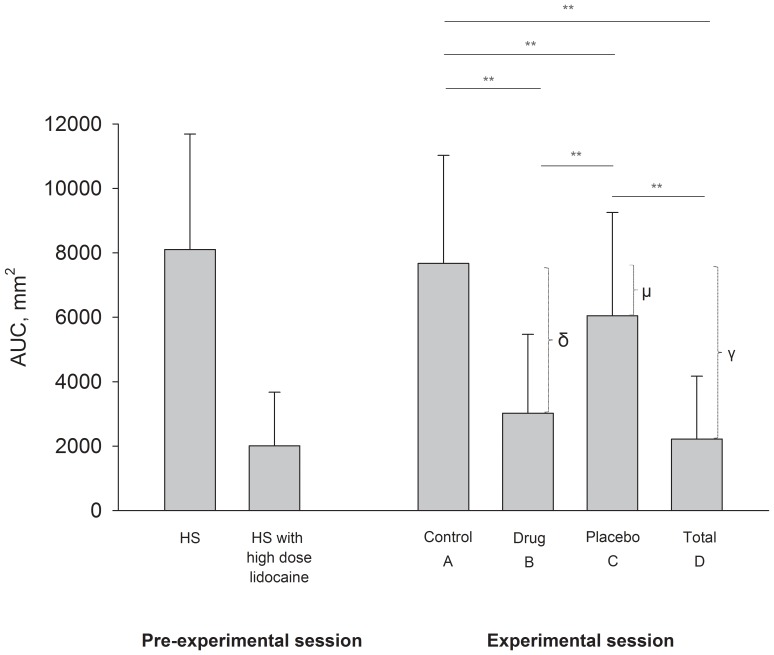
Pain intensity during sessions. Mean (SD) of area under the curve (AUC) of pain intensity in the pre-experimental session and in the conditions: control (A), drug (B), placebo (C), and total treatment (D) in the experimental session. The drug effect (δ) is the difference in pain between the control (A) and the drug (B) condition. The placebo effect (μ) is the difference in pain between the control (A) and the placebo (C) condition. The total treatment effect (γ) is the difference in pain between the control (A) and the total treatment (D) condition. * P<0.05, ** P<0.01.

Out of 48 included participants (50% male, 98% Caucasian, 94% students, mean age 23.4 (SD 6.2) years), 46 completed the study. Two participants withdrew consent; one because of high pain intensity and one could not be reached after the pre-experimental session. The AUC (mean and SD) in the pre-experimental session and in the experimental session are shown in Figure 2. There was a significant correlation between pain in the baseline condition and pain in condition A in the experimental condition (Pearson’s r = 0.60 AUC and 0.80 PP, P<0.001) and no difference in mean (P = 0.43 AUC and P = 0.19 PP) paired sample t-test (Figure 2). In the experimental session, a significant effect of treatment was observed (P<0.001, two-way ANOVA), and the placebo effect was statistically significant (mean AUC 1626 mm^2^ (95% CI, 938–2313), mean PP 10.5 (95% CI, 6.6–14.5) (P<0.001 for AUC and PP, A compared with C, paired t-test)) (Figure 2). Participants who had been conditioned during the pre-experimental session tended to have a larger placebo effect (conditioned group, n = 22, mean AUC 2120.0 (95% CI, 1122–3117) and mean PP 13.5 (95% CI, 6.5–20.4)) than the unconditioned group (n = 24, mean AUC 1173.1 (95% CI, 190–2156) and mean PP 7.9 (95% CI, 3.7–12.0)), although the difference was not statistically significant (P = 0.15 for AUC and P = 0.17 for PP, t-test). There was a positive correlation between the drug effect and the placebo effect (Pearson’s r = 0.33, P = 0.02).

### Primary Hypothesis

**Figure 3:**
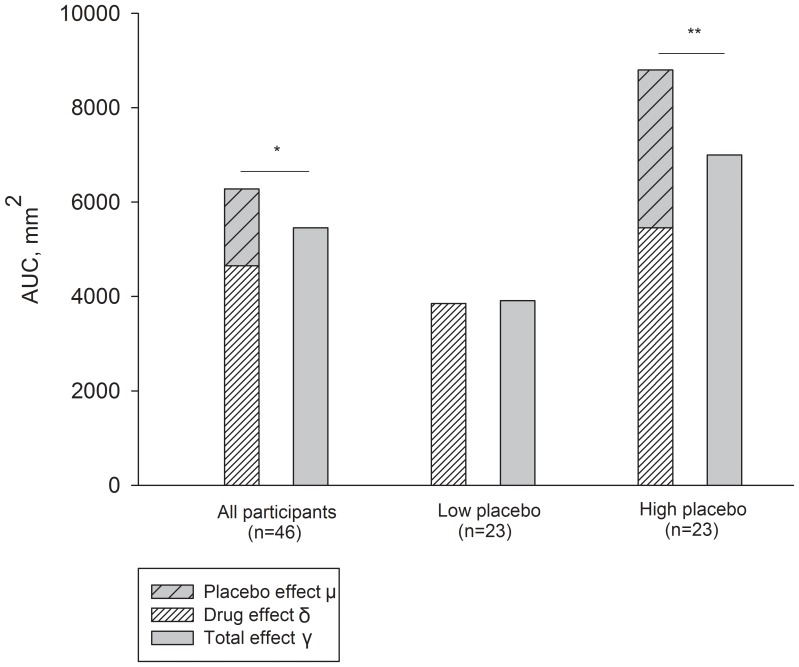
Subadditive placebo and drug effects. Mean area under the curve (AUC) for the sum of the drug effect and the placebo effect (δ+ μ) and for the total treatment effect (γ) for all participants and for the groups with low and high placebo effects.* P<0.05, ** P<0.01.

For the primary outcome, the AUC, a statistically significant difference was seen between the sum of the drug effect and the placebo effect (δ+μ) (mean AUC 6279 mm^2^ (95% CI, 4936–7622)) and the total treatment effect (γ) (mean AUC 5455 mm^2^ (95%CI, 4585–6324)) (P = 0.049, paired t-test) (Figure 3). The secondary outcome, the PP, showed a trend for the sum of the drug effect and the placebo effect (δ+μ) (mean PP 35.2 (95% CI, 27.0–43.3)) being larger than the total treatment effect (γ) (mean PP 30.0 (95% CI, 25.0–35.1)) (P = 0.065, paired t-test).

### Secondary Hypotheses

**Figure 4:**
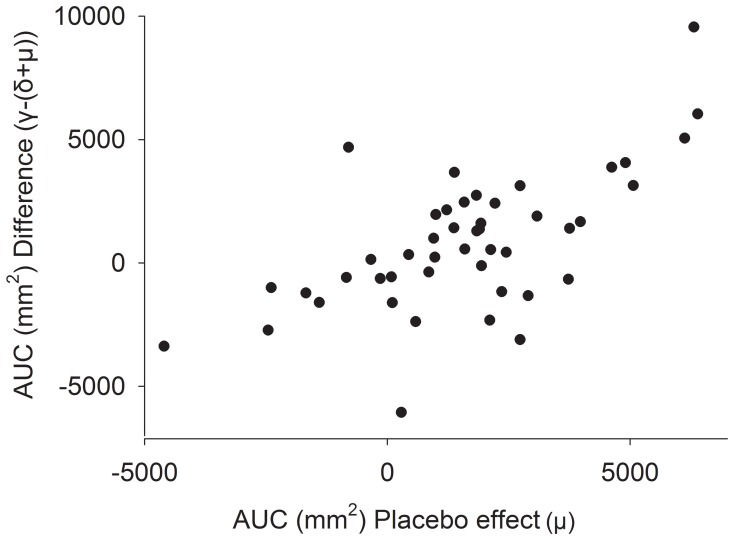
Correlation between placebo effects and the difference between total effect and the sum of drug and placebo effects. Correlation between the difference (between the total effect and the sum of the drug effect and the placebo effect) (γ-(δ+μ)) and the placebo effect (μ). Pearson’s r = 0.65, P = 0.006.

The difference between the total treatment effect and the sum of the drug effect and the placebo effect (γ-(δ+μ)) was larger in the 23 participants with the largest placebo effect (AUC 1804 mm^2^ (95% CI, 579–3030)) than in the 23 participants with the smallest or no placebo effect (AUC −155 mm^2^ (95% CI, −1178–868) (P = 0.015, t-test)). In addition, a statistically significant difference between the total treatment effect (γ) and the sum of the drug effect and the placebo effect (δ+μ) was seen only in the group with a large placebo effect (P = 0.006, paired t-test) and not in the group with a small placebo effect (P = 0.76, paired t-test) (Figure 3). There was a statistically significant correlation between the difference between the total treatment effect and the sum of drug effect and the placebo effect (γ-(δ+μ)) and the placebo effect (μ) (Pearson’s r = 0.65, P = 0.006; Figure 4). There was a tendency for the drug effect (δ) to be larger in the group with a large placebo effect than in the group with a small placebo effect (P = 0.089; Figure 3).

## Discussion

The main finding of the study was that the total treatment effect was smaller than the sum of the drug effect and the placebo effect. This finding rejects the assumption of additivity between the drug effect and the placebo effect. We also found that the difference between the total treatment effect and the sum of the drug effect and the placebo effect increased with increasing magnitude of the placebo effect. In meta-analyses that estimate treatment effects, it is assumed that if the placebo effect increases, the total treatment effect increases to the same degree. Our study thus refutes this assumption and suggests that the larger the placebo response in clinical trials, the larger the discrepancy between the actual drug effect and the estimated drug effect, i.e. the larger the underestimation of the drug effect size. This suggests that the placebo effect interacts with the drug effect in a way that is not independent of effect size and that placebo and drug effects are subadditive. Another factor that may contribute to the lack of additivity with large placebo effects is a ceiling effect, particularly if the drug effect is also large and the combined effect exceeds 100%. Although the placebo effect is only part of the placebo response in RCTs and despite the inherent relationship between a variable and a difference between that variable and another variable, this suggests that the additivity assumption in RCTs is also incorrect. The study supports the suggestions from RCTs that the larger the placebo response in clinical trials, the larger the discrepancy between the actual drug effect and the estimated drug effect, i.e. the larger the underestimation of the drug effect size [7]–[10]. The implication of this for clinical trials is that the drug effect sizes may tend to be underestimated, in particular in studies where the placebo response is large.

### Strengths and Limitations of the Study

To our knowledge, this is the first study using a within-subject balanced placebo design to estimate the drug effect, the placebo effect, and the total effect of analgesic treatment. A clear advantage of the balanced placebo design is that it estimates both the drug effect, the placebo effect, and the total treatment effect. This allows us to make a direct test of the additivity assumption. It is important to note, however, that the balanced placebo design does not estimate all components of the placebo response in clinical trials such as spontaneous remission of symptoms and other factors. In the present study, the placebo effect was optimised in various ways. First, the analgesic treatments were mixed in full view of the participants, which has been shown to influence the credibility of the treatment [21]. Second, in order to increase the variability of the placebo effect, the placebo manipulations were performed both with lidocaine conditioning (half of the participants) and with verbal suggestions (all participants). In line with previous studies [25]–[27], conditioning induced a slightly higher, albeit not statistically significant, placebo effect than verbal suggestions alone as well as an increased variability of the placebo effect. Third, in order to avoid or minimise response bias and negative expectations from giving the information that HS would induce pain, it was emphasised to the participants that the injections could cause very variable pain intensities depending on the exact site of the injection. However, it cannot be precluded that, for example, negative expectations of the ability of HS to cause pain may have attenuated the effect of the lidocaine in the hidden condition.

The pain model used in this study is a reliable pain model producing a stable pain of a moderate intensity for a limited time period. The technique is simple, safe, and easy to use, and the pain can be effectively treated using both open and hidden administrations. The limitations of the pain model and the BPTD are that they do not evaluate a clinically relevant chronic pain condition and that they examine the placebo effect rather than the placebo response. Also the within-subject comparison cannot be directly translated to RCTs using parallel group designs. If possible, these results should be confirmed in patients with a chronic pain condition, using also a between-group comparison and a larger sample size. However, there are several ethical problems in deceiving chronic pain patients in RCTs and practical problems in identifying a drug with consistent efficacy that is suitable for both hidden and open administration in a chronic pain population.

### Implications

The most important implication of the finding that drug effects and placebo effects are less than additive is that the drug effect can probably not be estimated by subtracting the placebo response from the total treatment effect in RCTs. Our study supports suggestions from clinical RCTs that the drug effect may be larger than the difference between the observed response during drug treatment and the observed response during placebo treatment, particularly in studies where the placebo response is large. Therefore RCTs are likely to underestimate the drug effect. Low assay sensitivity and failed trials are major issues in studies with a large and variable placebo response, e.g. trials of pain, depression, and cough [5], [11], [16], [17], [28], [29]. The present study confirms that a large placebo response in an RCT is likely to contribute much to assay sensitivity problems. Moreover, since several studies have shown that placebo responses are not stable across trials, variable placebo responses present a problem in estimating relative effect sizes in lack of direct comparison studies.

Although the present study assessed analgesic effects, its results may be applicable to other areas with a high placebo response. In fact, a recent study using a similar design showed that the combination of placebo and caffeine effects was larger than the total treatment effect. Although this finding was not statistically significant, it supports the notion that the additivity assumption is also invalid in trials of caffeine effects [30].

In traditional placebo trials using the open-hidden design, the placebo effect is estimated without the administration of inert treatments. The placebo component is defined as the difference between the open and the hidden administration of a drug treatment controlled for the natural history of the disease [31]. As this design is also based on the additivity model, it is likely that the open-hidden paradigm underestimates the placebo effects.

### Unanswered Questions and Future Research

The main concern raised in the present study is that drug effects may be underestimated in RCTs because of the possible lack of direct additivity. However, there are several other potential problems with RCTs, including questions about their validity [2]. Unintentional unblinding (e.g. due to side effects) may amplify non-specific effects in the drug treatment group but not in the placebo group [32], which challenges the internal validity of RTCs. This may be overcome by the use of active placebos, but this may also increase the response in the placebo group, and the effect size in such studies may not be comparable to that obtained in standard RCTs [33]. Different expectancies in placebo controlled trials (50% chance of receiving the drug) and clinical practice (100% chance of receiving the drug) question the external validity of such trials [34]. Although our study shows subadditivity of the placebo and the drug effect, we also found a positive correlation between placebo and drug effects. Attempts to decrease placebo effects may therefore also decrease drug effects. Placebo research has improved our knowledge of the underlying mechanisms, but many problems remain unsolved, and we need to critically interpret RCTs and to consider other designs.

While the balanced placebo design can be used in trials with healthy volunteers, it may be ethically more challenging to use it in clinical trials. In RCTs, patients consent to the fact that they may receive a drug or a placebo. In a balanced placebo trial design, however, patients would have to consent to this fact along with the fact that they will be deceived at some point in the trial. Crossover trials and studies using run-in placebo periods, direct comparison studies, and other designs have been considered and are presently being discussed [35], [36]. Until we are able to understand and control for placebo responses and improve trial designs, evidence-based medicine needs to take into account the magnitude of placebo responses in their interpretation of RCTs and meta-analyses.

## Conclusion

This experimental study supports suggestions from clinical trials that drug and placebo responses are less than additive. This may have important implications for the interpretation of RCTs, systematic reviews, and evidence-based clinical treatment guidelines because the drug effect may not be reliably assessed from randomised placebo controlled trials. Studies with large placebo responses may underestimate the true drug effect size. Future studies are needed to understand the impact of placebo responses in RCTs, and other designs to test the drug efficacy are wanted.
